# Prefrontal cortical activity during uneven terrain walking in younger and older adults

**DOI:** 10.3389/fnagi.2024.1389488

**Published:** 2024-05-03

**Authors:** Jungyun Hwang, Chang Liu, Steven P. Winesett, Sudeshna A. Chatterjee, Anthony D. Gruber, Clayton W. Swanson, Todd M. Manini, Chris J. Hass, Rachael D. Seidler, Daniel P. Ferris, Arkaprava Roy, David J. Clark

**Affiliations:** ^1^Department of Neurology, University of Florida, Gainesville, FL, United States; ^2^Department of Biomedical Engineering, University of Florida, Gainesville, FL, United States; ^3^McKnight Brain Institute, University of Florida, Gainesville, FL, United States; ^4^Department of Applied Physiology and Kinesiology, University of Florida, Gainesville, FL, United States; ^5^Brain Rehabilitation Research Center, Malcom Randall VA Medical Center, Gainesville, FL, United States; ^6^Department of Physical Therapy and Rehabilitation Sciences, Drexel University, Philadelphia, PA, United States; ^7^Department of Health Outcomes and Biomedical Informatics, University of Florida, Gainesville, FL, United States; ^8^Norman Fixel Institute for Neurological Diseases, University of Florida, Gainesville, FL, United States; ^9^Department of Biostatistics, University of Florida, Gainesville, FL, United States

**Keywords:** prefrontal cortical activity, fNIRS, walking, terrain unevenness, aging, mobility function

## Abstract

**Introduction:**

Walking in complex environments increases the cognitive demand of locomotor control; however, our understanding of the neural mechanisms contributing to walking on uneven terrain is limited. We used a novel method for altering terrain unevenness on a treadmill to investigate the association between terrain unevenness and cortical activity in the prefrontal cortex, a region known to be involved in various cognitive functions.

**Methods:**

Prefrontal cortical activity was measured with functional near infrared spectroscopy while participants walked on a novel custom-made terrain treadmill surface across four different terrains: flat, low, medium, and high levels of unevenness. The assessments were conducted in younger adults, older adults with better mobility function and older adults with worse mobility function. Mobility function was assessed using the Short Physical Performance Battery. The primary hypothesis was that increasing the unevenness of the terrain would result in greater prefrontal cortical activation in all groups. Secondary hypotheses were that heightened prefrontal cortical activation would be observed in the older groups relative to the younger group, and that prefrontal cortical activation would plateau at higher levels of terrain unevenness for the older adults with worse mobility function, as predicted by the Compensation Related Utilization of Neural Circuits Hypothesis.

**Results:**

The results revealed a significant main effect of terrain, indicating a significant increase in prefrontal cortical activation with increasing terrain unevenness during walking in all groups. A significant main effect of group revealed that prefrontal cortical activation was higher in older adults with better mobility function compared to younger adults and older adults with worse mobility function in all pooled terrains, but there was no significant difference in prefrontal cortical activation between older adults with worse mobility function and younger adults. Contrary to our hypothesis, the older group with better mobility function displayed a sustained increase in activation but the other groups did not, suggestive of neural compensation. Additional findings were that task-related increases in prefrontal cortical activation during walking were lateralized to the right hemisphere in older adults with better mobility function but were bilateral in older adults with worse mobility function and younger adults.

**Discussion:**

These findings support that compared to walking on a flat surface, walking on uneven terrain surfaces increases demand on cognitive control resources as measured by prefrontal cortical activation.

## 1 Introduction

Successful mobility function in the home and community settings requires walking in complex environments. A common form of complex walking is uneven terrain, which may include varying heights, textures, and compliance of the walking surface. A considerable amount of evidence shows that walking in complex environments requires cognitive control processes including attention and planning of motor actions (Yogev-Seligmann et al., 2008). Such environments create a potential risk for older adults, who may show declines in function across multiple systems, including sensation, cognition, and mobility (Mahncke et al., 2006). Control of complex walking tasks may be compromised by limited cognitive resources especially in the prefrontal cortex, thereby affecting the quality of movement (Clark, 2015). The prefrontal cortex, which predominantly supports cognitive processes, exhibits declines with age, potentially leading to further compromises in motor control during complex walking tasks (Seidler et al., 2010). Brain over-activation during a complex walking task may serve as a potential risk factor for an elevated risk of falls (Verghese et al., 2017) and an increased fear of falling (Holtzer et al., 2019).

Many studies of cognitive aging have demonstrated that as task difficulty increases during task performance, there is a corresponding increase in brain activity (Mattay et al., 2006; Cappell et al., 2010). This type of study has been conducted in the context of complex (cognitively engaging) walking tasks, examining various experimental contexts such as walking while performing a secondary cognitive task (Holtzer et al., 2011; Ohsugi et al., 2013; Beurskens et al., 2014; Fraser et al., 2016) and stepping over obstacles (Maidan et al., 2018; Nordin et al., 2019; Chatterjee et al., 2020). However, there is a gap in the literature because these studies have not been conducted under the same experimental context involving parametric manipulations of task difficulty while walking. It is important to investigate whether systematically adjusting the complexity of a walking task will result in corresponding increases in brain activity, and how this relationship may be affected by aging and by mobility function. This insight will help to support the future development of outcome measures and intervention strategies aimed at studying and improving complex walking function in older adults.

Functional near-infrared spectroscopy (fNIRS) has emerged as a hemodynamic-based neurophysiological approach, particularly adept at assessing brain activity during walking (Herold et al., 2017). This is attributed to its portability and tolerance for motion as compared to functional magnetic resonance imaging (fMRI) (Scarapicchia et al., 2017). One particularly robust effect found in many fNIRS studies of walking is that compared with younger individuals, older adults often exhibit heightened prefrontal cortical activation during the performance of dual-task walking (Holtzer et al., 2011; Ohsugi et al., 2013; Beurskens et al., 2014; Fraser et al., 2016) and obstacle crossing (Mirelman et al., 2017; Hawkins et al., 2018; Chatterjee et al., 2020). Increased brain activity coupled with preserved behavioral performance is typically interpreted as compensation (Fettrow et al., 2021b).

The compensation-related utilization of neural circuits hypothesis (CRUNCH) (Reuter-Lorenz and Cappell, 2008) can provide a framework for interpreting brain activity during walking in older adults. The CRUNCH model proposes that greater cortical activity occurs during task performance as task difficulty increases. This greater cortical activity can include higher amplitude activation within a specific region and/or recruitment of additional regions. At lower levels of task demand, older adults tend to recruit more neural resources, leading to heightened cortical activity compared with younger adults. This suggests that compensatory recruitment is needed to perform the task well. At higher task demands, this compensatory strategy may become ineffective due to the saturation of cognitive load capacity. At this point, older adults may exhibit reduced cortical activity, reaching their load capacity sooner than their younger counterparts (Schneider-Garces et al., 2010). However, brain activity in older adults during walking is not consistent in the literature, as there have been reports of over-activation (Mirelman et al., 2017; Hawkins et al., 2018; Chatterjee et al., 2020; Nobrega-Sousa et al., 2020), under-activation (Beurskens et al., 2014; Baek et al., 2023) or no change (Fraser et al., 2016; Rosso et al., 2024). Brain over-activation and under-activation appear to depend on the type of walking task and more specifically on the difficulty of that task. Utilizing task paradigms that systematically manipulate multiple levels of load is crucial for understanding the relationship between cognitive load and brain activity, as proposed by the CRUNCH model (Reuter-Lorenz and Cappell, 2008). However, the CRUNCH framework has not yet been applied to a complex walking task with parametrically varying levels of task difficulty.

In the present study, we used four levels of task difficulty to investigate whether brain activation differs while younger and older adults walk on varying levels of terrain unevenness. The use of multiple levels of task difficulty enables assessment of whether prefrontal cortical activation increases systematically with increased task demands, and whether there are age and functional differences in resource ceiling levels (Fettrow et al., 2021b). Compared with dual-task walking paradigms, which distract participants cognitively and intermittently (Smith et al., 2016; Ramirez and Gutierrez, 2021), walking over uneven terrain requires a continuous cognitive load directed at the control of the walking task without task switching (Downey et al., 2022; Liu et al., 2024). To our knowledge, age-related changes in brain activation have not been investigated in participants walking across varying levels of terrain unevenness.

We evaluated changes in three groups of participants: younger adults, older adults with better mobility function, and older adults with worse mobility function. We also assessed the extent to which task-related prefrontal cortical activation fits the CRUNCH framework. Our main hypotheses were that (a) prefrontal cortical activation would increase in all participant groups as terrain unevenness increased; (b) older adults with worse mobility function would exhibit greater prefrontal cortical activation at lower levels of terrain unevenness, consistent with compensatory recruitment of the prefrontal networks in the older brain; and (c) older adults with worse mobility function would reach a plateau in prefrontal recruitment before younger adults as reflected by a plateau or reduction of prefrontal cortical activation at higher levels of terrain unevenness.

## 2 Materials and methods

### 2.1 Participants

This study is part of the larger Mind in Motion project (Clark et al., 2019), which investigates the neural control of mobility in older adults. Here, we analyzed data from 65 participants (37 female): 22 healthy younger adults and 43 community-dwelling older adults. All participants provided written consent, and the protocol, approved by the Institutional Review Board at the University of Florida, followed ethical guidelines. Briefly, inclusion criteria comprised age ranges of 20–40 years for younger adults and ≥ 65 years for older adults, and the ability to walk 400 m in <15 min without assistance (Vestergaard et al., 2009). Exclusion criteria involved the presence of mild cognitive impairment (i.e., Montreal Cognitive Assessment [MoCA] score < 26) (Nasreddine et al., 2005) and other significant existing medical conditions or historical health issues that could impede the execution of the uneven terrain walking task. Full inclusion and exclusion criteria have been described previously (Clark et al., 2019). Older adults were categorized into their respective mobility function groups based on their Short Physical Performance Battery (SPPB) scores, with SPPB ≥10 indicating better mobility function and SPPB <10 indicating worse mobility function (Pavasini et al., 2016). Details of the SPPB test are provided in section 2.3.1.

### 2.2 Overall design and procedures

Our cross-sectional study involved three separate visits occurring within a 30-day span. During the initial visit, participants underwent baseline anthropometric, motor, and cognitive assessments. Additionally, participants performed an overground terrain walking task to measure their preferred speed; this was used to individualize the treadmill walking speed. The second visit utilized a specialized terrain surface on a standard exercise treadmill, which we developed for the study (Clark et al., 2019; Downey et al., 2022). In this visit, we employed fNIRS to assess changes in prefrontal cortical activation as participants walked on the treadmill across four levels of terrain unevenness, including flat, low, medium, and high. A few weeks after the fNIRS visit, we acquired a T1-weighted MRI scan to gather information on the distance from the fNIRS optodes to the cortical surface. The timing of the T1 scan acquisition relative to the study was chosen to optimize logistical considerations and resource availability.

### 2.3 Baseline assessments

#### 2.3.1 Mobility function assessment

To classify older adults into better or worse mobility function, we utilized the SPPB test, which incorporates a 4-meter usual pace walk, time to complete five unassisted chair stands, and three standing balance assessments (Guralnik et al., 1994). To evaluate walking ability, we employed the 400-meter walk test (Vestergaard et al., 2009) to determine if participants could complete the test within 15 min. To assess participants’ balance confidence, we utilized the Activities-specific Balance Confidence Scale (ABC Scale), a structured questionnaire designed to measure self-perceived confidence level during various activities and situations (Powell and Myers, 1995).

#### 2.3.2 fNIRS optodes to cortical surface distance measurement

Age-related alterations in brain volume and cortical thickness, including prefrontal regions (Dotson et al., 2015), may alter the distance between the fNIRS optodes and the cortical tissue, potentially introducing inter-individual variability into the fNIRS amplitude and spatial resolution (Chen et al., 2020). To attempt to correct for these potential variations, we measured the distance from fNIRS optodes to the cortical surface in each participant using T1-weighted MRI images (Liu et al., 2024). This measure was then integrated as a covariate to adjust task-dependent prefrontal cortical activation. The parameters of the MRI images were: repetition time (TR) = 2000 ms, echo time (TE) = 2.99 ms, flip angle = 8°, voxel resolution = 0.8 mm^3^, field of view = 256 × 256 × 167 mm^2^ (4,22 min of scan time), using a 64-channel coil array on a 3 T Siemens MAGNETOM Prisma MR scanner (Liu et al., 2024). Using the 2020 version of the Statistical Parametric Mapping (SPM12) software, we normalized all participant T1 scans using the DARTEL algorithm with standard parameters (Ashburner, 2007). In this MNI space, we could then register the fNIRS optode location on the scalp for each participant. Recognized international marker locations for the F3 and F4 optodes were translated into MNI coordinates as (−42.6, 58.0, 39.6) and (46.9, 56.7, 40.2), respectively (Okamoto et al., 2004). To integrate these coordinates with each T1 scan, dots were generated at the known F3 and F4 MNI xyz coordinates, maintaining a fixed radial distance of 2 mm, using MRI viewing software MRIcrogl (Rorden and Brett, 2000). Those dots were saved as a separate volumes, with a copy created for each participant under analysis. The normalized T1 scans and the electrode location volume were then reverted to each participant’s space utilizing SPM12’s normalize-write option with the same parameters as the forward warp but employing the inverse deformation field. Once the electrode location volume was registered in the individual’s space for each participant, overlays were generated in ImageJ, comprising the native T1 scan and the inverse warp of the electrode location volume (Schneider et al., 2012). With a known radial dot size of 2 mm, this measurement was employed to scale the images in ImageJ, and a line was drawn from the dot to the cortex surface in the T1 scan using ImageJ’s straight-line tool. The length of the drawn line was subsequently measured based on the prior scaling, providing the distance from the optode location on the scalp to the brain for further analysis. This procedure was replicated for all participants, and the distance measurements were recorded for subsequent use as covariates in fNIRS analysis.

### 2.4 Experimental protocol

#### 2.4.1 Terrain unevenness design

Four walking terrain surfaces (flat, low, medium, high) were designed, each presenting distinct levels of terrain unevenness (Clark et al., 2019; Downey et al., 2022). Terrain unevenness was manipulated using rigid foam disks (non-compressible, each 12.7 cm in diameter; Blockwire Manufacturing LLC, Goshen, AL, USA) that varied in height. The low terrain level of unevenness consisted entirely of 1.3 cm tall disks painted in yellow. The medium terrain level comprised 50% 1.3-cm tall and 50% 2.5-cm tall disks painted in orange. The highest and most challenging terrain unevenness level included three different height disks painted in red: 50% at 3.8 cm, 20% at 2.5 cm, and 30% at 1.3 cm. For the easiest terrain level, the flat terrain had no disks on the walking surface; instead, green circles were painted on the mat and treadmill belt. This was done to ensure that the visual aspect of the flat condition was similar to the other terrain conditions.

#### 2.4.2 Overground version of terrain walking

3.5 m mats with the same terrain surfaces were created for the overground walking assessments. Participants walked over these four surfaces so that we could determine their preferred overground walking speeds. Participants were directed to walk at a natural, comfortable pace over each terrain level a total of three times. The time to complete the middle 3-m portion of the mat was measured with a stopwatch. We calculated the mean over-ground walking speed for each terrain and then multiplied the participant’s slowest average over-ground speed (slowest terrain) by 75% to determine the target treadmill speed for that participant to use in the treadmill tests. This speed remained constant across all terrains during treadmill walking. This was done to accommodates participants with different levels of physical function (Clark et al., 2019) and to ensure that terrain is the sole factor that differs across conditions, rather than also having possible differences in walking speed, which could affect brain activity (Liu et al., 2024).

#### 2.4.3 Treadmill version of terrain walking

**Figure 1:**
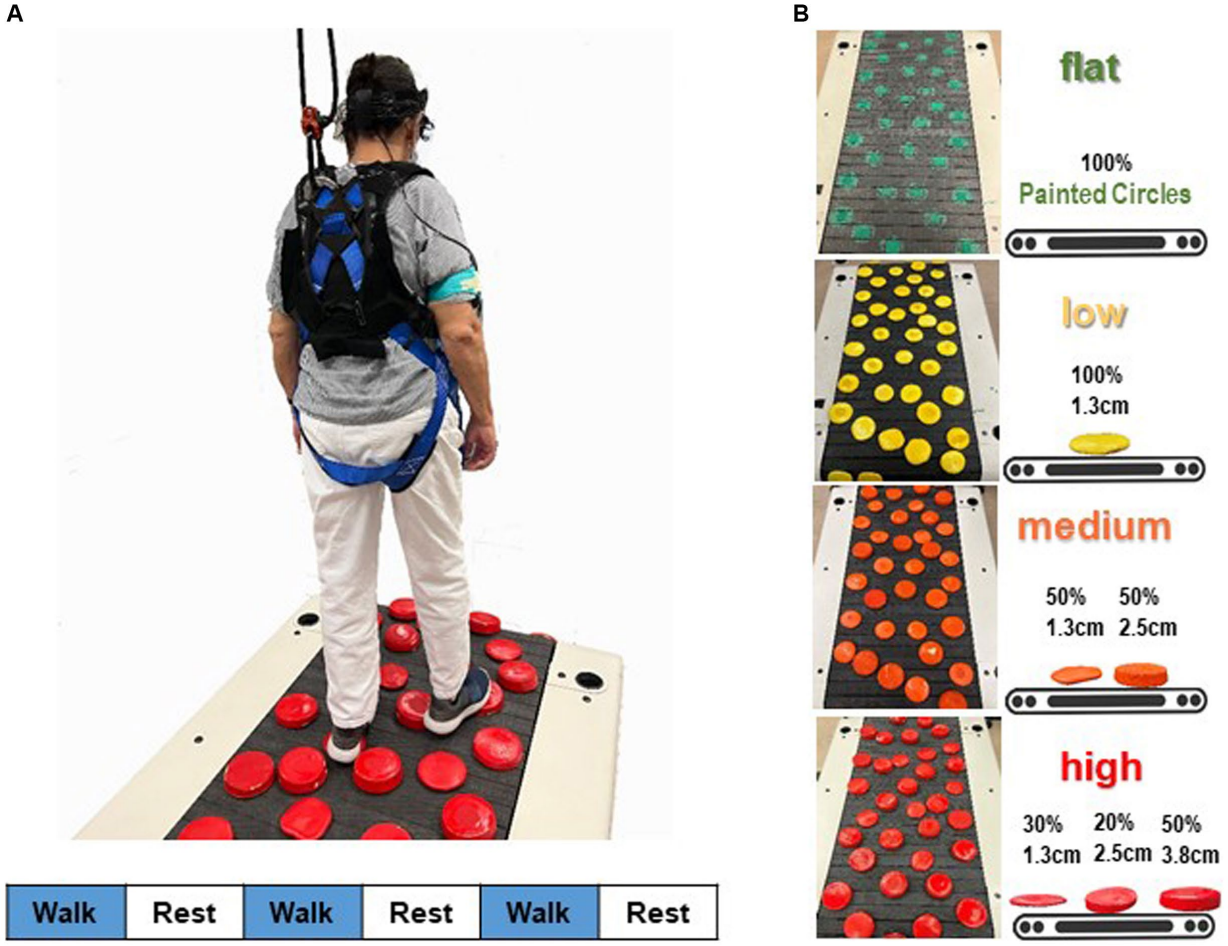
Schematic overview of the fNIRS measurement during walking. **(A)** Participants’ prefrontal cortical activation was recorded while they walked on a terrain treadmill belt. A block design, in which three 30-s active blocks of walking were alternated with three 30-s reference blocks (standing quietly), was used to assess prefrontal cortical activation during walking. Stimulus event onsets are shown in the lower part of the figure. Blue indicates walking on the treadmill (handrail-not supported; active condition); white, standing on the treadmill surface (handrail-not supported; reference control condition). **(B)** Four terrain surfaces were generated by varying the height of the obstacles in the terrain. Percentages refer to the proportion of disks that were the specified height.

Figure 1 illustrates the adjustment of terrain unevenness of the treadmill walking task. Disks were applied to the walking surface of a slat belt treadmill (PPS 70 Bari-Mill, Woodway, Waukesha, WI, USA; 70 cm x 173 cm walking surface) using self-adhesive hook-and-loop fasteners. This setup allowed for easy interchangeability of disks between different terrain conditions and took only a few minutes. The spatial arrangement of the terrain disks remained consistent across four terrain surfaces (Downey et al., 2022). We arranged the layout to prevent large gaps and adjacent disks, minimizing participants’ chances of finding an easy spot to step. Each level of terrain unevenness was utilized in a separate walking trial, with the order of unevenness conditions randomized. For safety, participants wore a harness secured to an overhead rail (Portable Track System, Solo-Step, North Sioux City, SD, USA), designed to catch them in case of a trip or fall without disrupting natural walking movements. Research staff members were positioned behind and to the sides of the treadmill to prevent accidental straying. Treadmill handrails were removed to discourage holding for support, though participants could briefly grab a staff member’s arm in case of stumbling. Participants were instructed to release the support quickly and resume normal walking. Participants could hold a staff member’s arm for support during acceleration or deceleration but had to walk independently before the trial officially began. All participants completed their walking trials unaided, and there were no instances of arm grabbing during the steady-state walking when the measurements were taken. Therefore, no data on arm grabbing was recorded.

### 2.5 fNIRS

#### 2.5.1 fNIRS setup

**Figure 2:**
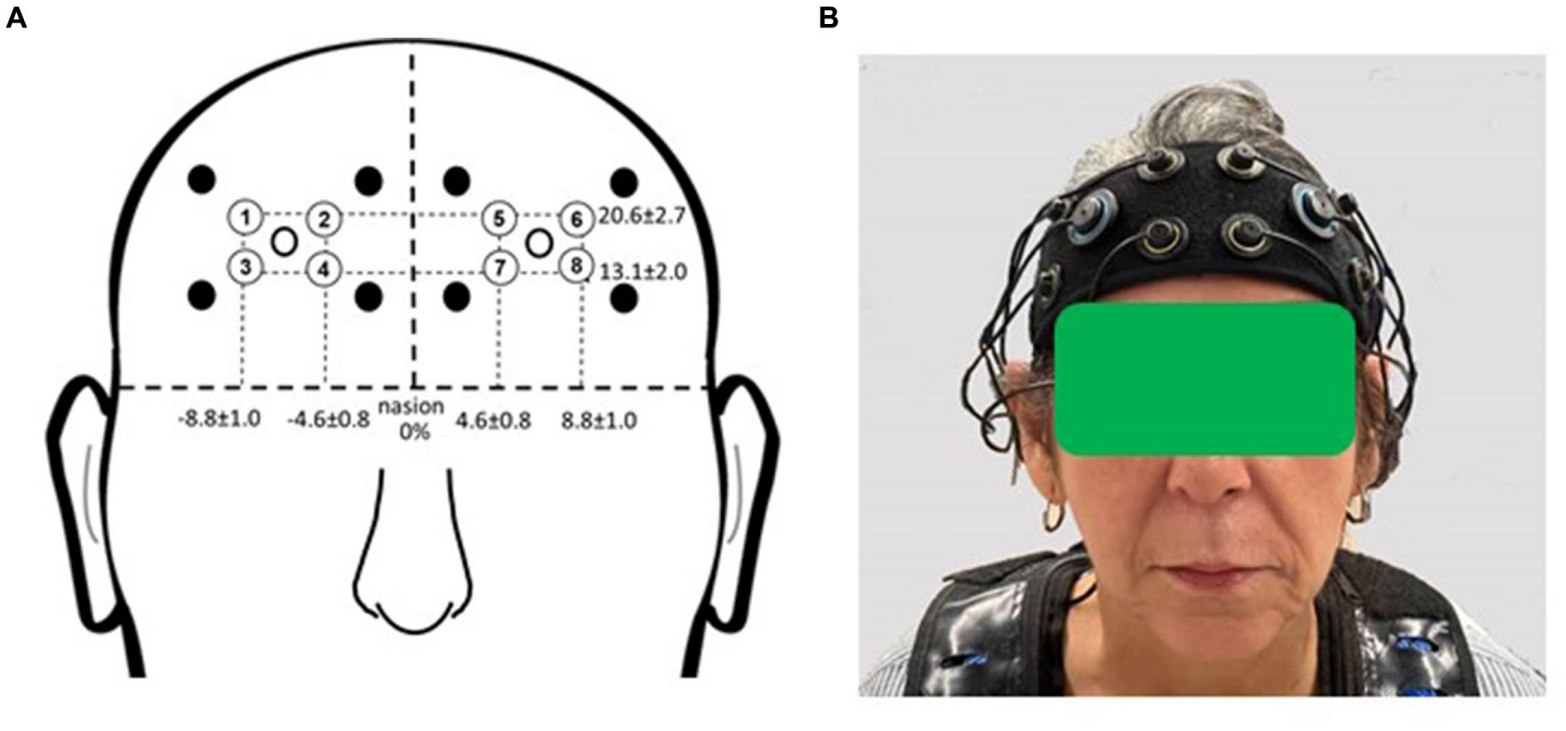
fNIRS recording sites. **(A)** Group mean fNIRS recording sites expressed as a percentage of head circumference (lateral direction) and nasion to inion distance (vertical direction). Light emitters are shown as closed circles and light detectors as open circles. The recording site (channel) is estimated as halfway between each emitter-detector pair and is shown with a circled number. Circled numbers indicate the recording site (channel number). **(B)** Placement of the fNIRS headband device on the participant’s head. Informed consent was obtained for use of this image.

Participants were outfitted with a commercially available multichannel continuous-wave fNIRS unit (OctaMon, Artinis Medical Systems, Nijmegen, Netherlands) to measure prefrontal cortical activation during the experimental period (Figure 2). The headband, containing light sources emitting near-infrared light at continuous wavelengths of 760 nm and 850 nm, along with two near-infrared light detectors, was worn by participants. Separate recording channels were distinguished by time-division multiplexing. The headband was positioned just above the eyebrows, with its midline aligned with the midline of the face. The source-detector optode location on the headband was fixed at 3.5 cm. Anatomical recording sites for each channel were estimated by measuring the mid-point location between each light emitter-detector pair, reported in reference to the International 10–10 system (Koessler et al., 2009). Horizontal placement in the transverse plane was measured as a percentage of head circumference, and vertical placement in the sagittal plane as a percentage of the nasion to inion distance. The group mean recording sites relative to the nasion were as follows for the horizontal and vertical directions, respectively: 20.6% ± 2.7 and 13.1% ± 2.0 (for the medial optodes); 4.6% ± 0.8 and 8.8% ± 1.0 (for the lateral optodes). The medial left and right fNIRS optodes were approximately aligned with the landmarks of Fp1 and Fp2. The lateral left and right optodes were approximately aligned with the landmarks of AF7 and AF8. These measurement locations correspond to the medial and lateral subregions of Brodmann Area 10 (Ben Mahmoud et al., 2009).

#### 2.5.2 fNIRS data acquisition

We collected fNIRS data while participants walked on the treadmill featuring four different terrain surfaces. Employing a block design, we alternated three 30-s active blocks of walking with three 30-s reference blocks. In the active blocks, treadmill speed gradually increased to reach each participant’s target speed. Once the target speed was attained, the walking trial officially commenced. During the reference blocks, participants stood still and remained silent on the treadmill (Holtzer et al., 2016). For this task, participants completed three pairs of reference/active blocks for each terrain condition (i.e., a total of 12 pairs). Start and end points of each block were manually marked using a wireless remote device (PortaSync, Artinis Medical Systems, Nijmegen, Netherlands), which placed event markers in a separate recording channel that was later time-synchronized to the fNIRS signals. The data were sampled at 10 Hz and exported to a computer for analysis. The order of the four terrains was pseudorandomized (Downey et al., 2022). For instance, if the first terrain was flat or low, then the second terrain was medium or high, and vice versa.

#### 2.5.3 Behavioral data acquisition

During the 30-s active blocks in each walking trial for every terrain, the number of steps was recorded using a handheld tally counter. To assess walking cadence variability, we computed the standard deviation and mean of the number of steps across the four terrains for each participant. The coefficient of variation for walking cadence variability was calculated by dividing the standard deviation by the mean of the data and then multiplying by 100 to express it as a percentage. Immediately after each terrain, participants provided subjective ratings of their self-perceived stability using a modified version of the Rate of Perceived Stability scale (Downey et al., 2022). The modified scale comprises five distinct ratings, spanning from steady to very unbalanced. Participants responded to a series of questions to assess their walking experience, beginning with, “Did it feel like work to keep your balance?” If the answer was yes, a stability rating was assigned; if no, the next question was asked.

#### 2.5.4 fNIRS data processing

We utilized a differential pathlength factor value of 6 in our fNIRS data analysis. Prefrontal oxyhemoglobin (O2Hb) concentrations were computed following the modified Beer–Lambert Law and analyzed using custom MATLAB programs (version R2015a, MathWorks, Natick, MA, USA). Raw fNIRS signals underwent preprocessing, including detrending and application of a low-pass filter with a cutoff frequency at 0.14 Hz to minimize physiological noise (Holtzer et al., 2011). A wavelet filter was employed to mitigate the impact of motion artifacts (Herold et al., 2018). Subsequently, a trained team member visually inspected the data, excluding any channels with evident signal quality issues (e.g., high amplitude artifacts inconsistent with physiological activity or no apparent change in signal). We selected O2Hb values for characterizing the tasks due to their greater reliability and sensitivity to walking-related cerebral blood flow compared with deoxyhemoglobin values (Miyai et al., 2001). Task-related changes in prefrontal cortical O2Hb (∆O2Hb) were calculated for each participant and terrain. This involved averaging the three blocks of active O2Hb (walking) and three blocks of resting O2Hb (standing), followed by computing the task-related change using the formula: ∆O2Hb = active O2Hb − reference O2Hb. The ∆O2Hb data from all channels were averaged for each participant and each terrain before subsequent analyses.

### 2.6 Statistical analysis

The normality assumption for the data was assessed through histograms, quantile-quantile plots, and the Shapiro–Wilk test, with all variables showing *p* > 0.05, indicating normal distribution. Baseline characteristics and cognition/mobility assessments underwent one-way analysis of variance for continuous data and chi-square (χ^2^) tests for categorical data. Additionally, one-way analysis of variance was utilized to assess differences in walking cadence variability among groups. A linear mixed model guided analysis investigated the impact of group (younger adults, older adults with better mobility function, and older adults with worse mobility function) as a between-subject factor, terrain (flat, low, medium, and high surfaces) as a repeated within-subject factor, and the interaction between group and terrain on prefrontal cortical activation (the mean of all 8 channels), overground walking speed, and walking cadence. The model of task-related prefrontal cortical activation included treadmill walking speed and the mean distances from the fNIRS F3 and F4 optodes to the cortical surface as separate covariates. Distinct models were employed to evaluate differences in prefrontal cortical activation based on hemisphere and channels. This included comparisons between the right side (the mean of channels 1, 2, 3, and 4) and the left side (the mean of channels 5, 6, 7, and 8), as well as comparisons across four individual channels for each side. If the group by terrain interaction achieves statistical significance, pairwise *post hoc* tests were conducted between groups for each terrain separately or between terrains for each group separately. However, in the absence of a significant interaction, the main effects of terrain and group were assessed independently for all combinations of terrains and groups. Pairwise *post hoc* tests were adjusted for multiple comparisons using the false discovery rate (FDR) correction method. We also calculated partial Eta squared (η_p_^2^) as a measure of effect size for the main effects and used the following definitions for the effect sizes: 0.01 = small, 0.06 = medium, and 0.14 = large effect size (Lakens, 2013). Data from two younger adults and one older adult with worse mobility function were excluded due to a malfunction in the fNIRS system during data collection. Additionally, due to unforeseen circumstances, a small amount of behavioral data (i.e., the number of steps) was not recorded and therefore was not used in the final analysis. For the perceived stability outcome measure, an ordinal logistic regression model was employed, as the values are derived from a discrete set of ordered ratings (i.e., steady, unsteady, mildly, moderately, and very unbalanced). All statistical analyses were conducted using JMP software (JMP® 15.0, SAS Institute Inc., Cary, NC, USA), except for the ordinal logistic regression analysis, which was performed in Stata (16.1, StataCorp LLC, College Station, TX, USA). The significance level (α) was set at 0.05.

## 3 Results

### 3.1 Participant characteristics

Table 1 presents the participant characteristics. The study included a total of 65 participants, with 22 younger adults, 29 older adults with better mobility function, and 14 older adults with worse mobility function. Older adults with better mobility function had a mean SPPB score of 11.0, while older adults with worse mobility function had a mean score of 8.0. Age did not significantly differ between the two older groups (*p* = 0.674). Sex distribution was equal in each group (χ^2^ = 3.82, *p* = 0.148), with 60 participants being right-handed (χ^2^ = 1.81, *p* = 0.771). No older participant showed signs of cognitive impairment (MoCA >26) or walking disability (all completed a 400-m walk in <15 min). Older adults with worse mobility function exhibited a higher body mass index compared with younger adults (*p* < 0.001) and older adults with better mobility function (*p* = 0.003). In comparison to younger adults, both older adults with better mobility function and older adults with worse mobility function reported lower balance confidence score (*p* = 0.012 and *p* < 0.001, respectively) and lower SPPB score (*p* < 0.001 and *p* < 0.001, respectively), while older adults with worse mobility function exhibited longer completion times for the 400-m walk (*p* < 0.001). Older adults with worse mobility function, when compared with older adults with better mobility function, exhibited lower balance confidence score (*p* < 0.001) and lower SPPB score (*p* < 0.001), and longer completion times for the 400-m walk (*p* = 0.003). Older adults with worse mobility function showed greater distances from fNIRS F3 and F4 optodes to the cortical surface compared with younger adults (*p* < 0.001 and *p* < 0.001, respectively) and older adults with better mobility function (*p* = 0.001 and *p* < 0.001, respectively). Furthermore, there was no significant difference in distances from either fNIRS F3 (*p* = 0.081) or F4 (*p* = 0.052) optodes to the cortical surface between younger adults and older adults with better mobility function.

**Table 1:** Participant characteristics (Mean ± SD presented).

	Younger adults (Y)		Older adults Better mobility function (BM)		Older adults Worse mobility function (WM)		Pairwise comparison		
							Y vs. BM	Y vs. WM	BM vs. WM
*N*	22		29		14				
Sex (M/F)	10/12		15/14		3/11				
Hand dominance (Left/Right)	1/21		3/26		1/13				
Age (years)	22.8	±3.5	74.0	±4.9	74.7	±6.8	<0.001	<0.001	0.674
Weight, kg	67.5	±12.7	75.2	±13.7	81.6	±14.7	0.078	0.011	0.154
Height, cm	169.8	±11.7	171.3	±10.7	165.9	±6.2	0.606	0.420	0.352
Body Mass Index, kg/m^2^	23.3	±3.2	25.6	±3.6	29.7	±5.4	0.052	<0.001	0.003
SPPB, score	12.0	±0.0	11.0	±0.9	8.0	±0.8	<0.001	<0.001	<0.001
400 m walk, min	5.8	±0.8	6.0	±1.1	6.9	±1.0	0.439	< 0.001	0.003
ABC scale, score	98.1	±1.9	91.4	±8.3	80.5	±14.5	0.012	<0.001	<0.001
MoCA cognitive test, score	28.0	±1.6	27.1	±1.5	27.3	±1.6	0.117	0.239	0.728
Distance from fNIRS F3 optodes to cortical surface, mm	13.8	±4.6	14.7	±3.7	19.9	±3.4	0.081	<0.001	0.001
Distance from fNIRS F4 optodes to cortical surface, mm	12.9	±4.0	13.7	±3.8	18.7	±1.8	0.052	<0.001	<0.001

SD, standard deviation; SPPB, Short Physical Performance Battery; ABC, Activities-specific Balance Confidence; MoCA, Montreal Cognitive Assessment; fNIRS, Functional near-infrared spectroscopy; Y, younger adults; BM, older adults with better mobility function; and WM, older adults with worse mobility function.

### 3.2 Overground walking speed for customizing treadmill walking speed

**Figure 3:**
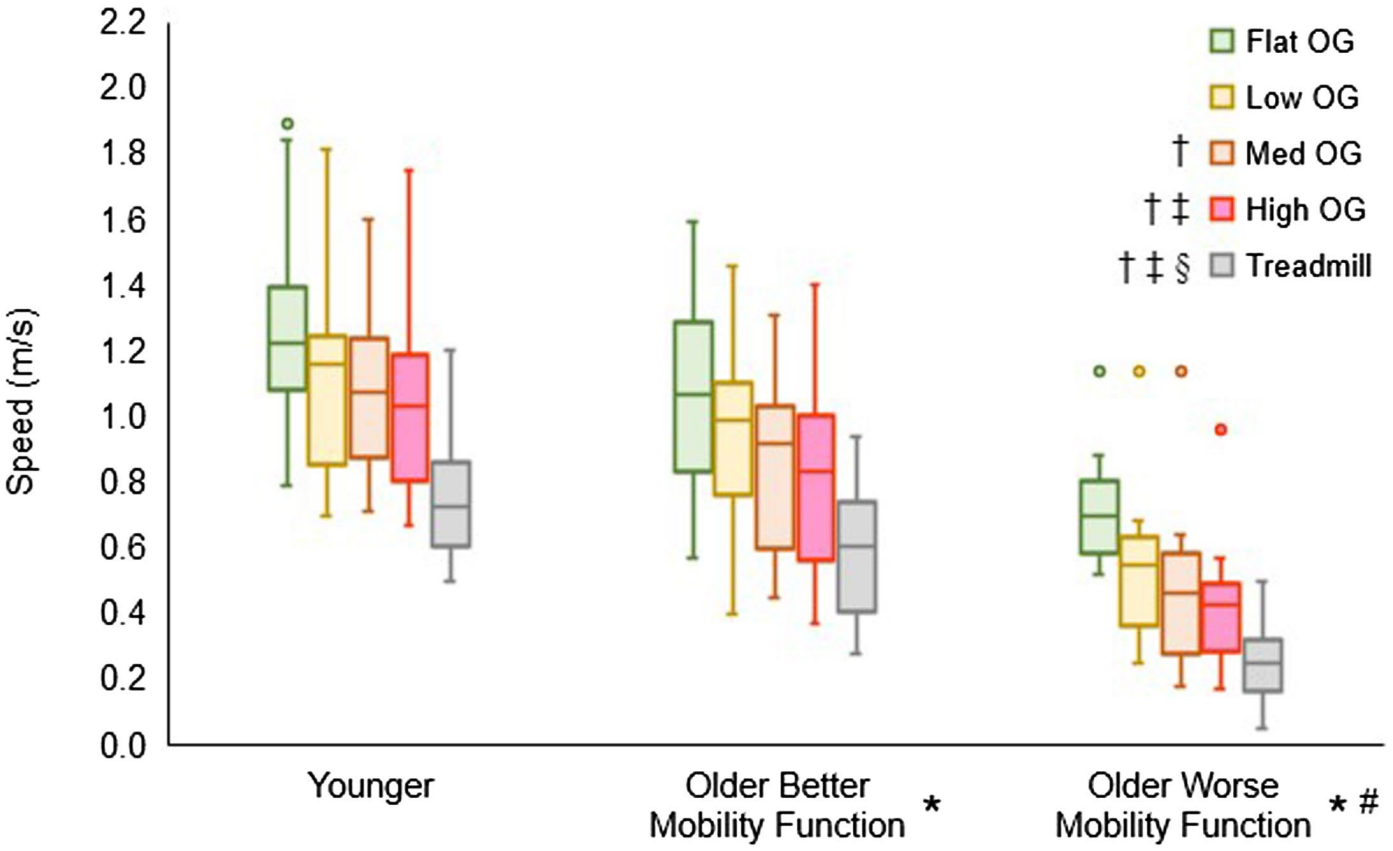
Box plots illustrating the distribution of overground (OG) walking speed and treadmill speed calculations. Unfilled circles denote outliers. † Represents a significant decrease in speed versus flat terrain, ‡, a significant decrease in speed versus low terrain; §, a significant decrease in speed versus medium terrain; *, a significant decrease in speed versus younger adults for overground walking speed and treadmill walking speed; and #, a significant decrease in speed versus older adults with better mobility function for overground walking speed and treadmill walking speed.

Figure 3 displays the overground walking speeds of participants for each terrain, categorized by group, alongside the customized treadmill walking speed for each group. The group by terrain interaction did not reach statistical significance (*F*_6, 237_ = 0.55, *p* = 0.770, η_p_^2^ = 0.02), suggesting that the impact of task difficulty remained consistent across groups. However, a significant main effect of terrain was observed (*F*_3, 237_ = 63.06, *p* < 0.001, η_p_^2^ = 0.84), indicating a significant difference among terrains in all pooled groups. *Post hoc* tests revealed significantly slower speeds for low (*p* < 0.001), medium (*p* < 0.001), and high (*p* < 0.001) terrains compared with flat terrain. Additionally, medium (*p* < 0.001) and high (*p* < 0.001) terrains exhibited slower speeds compared with low terrain, and high terrain (*p* = 0.012) showed slower speeds compared with medium terrain. Furthermore, the main effect of group yielded significant results (*F*_2, 237_ = 18.96, *p* < 0.001, η_p_^2^ = 0.17), indicating a significant difference among groups in all pooled terrains. *Post hoc* tests indicated that both older adults with better mobility function (*p* = 0.014) and older adults with worse mobility function (*p* < 0.001) walked slower than younger adults. In addition, older adults with worse mobility function walked slower than their counterparts with better mobility function (*p* < 0.001). In a separate analysis, treadmill walking speed (mean ± standard deviation), calculated from overground walking speeds, was significantly slower in both older adults with better mobility function (0.57 ± 0.18 m/s; *p* < 0.001) and older adults with worse mobility function (0.26 ± 0.11 m/s; *p* < 0.001) compared with younger adults (0.73 ± 0.17 m/s). Additionally, older adults with worse mobility function exhibited a slower walking speed than those with better mobility function (*p* < 0.001). Descriptive data is available in Supplementary Table S1.

### 3.3 Task-related prefrontal cortical activation

#### 3.3.1 Prefrontal cortical activation (ΔO2Hb) during terrain walking

**Figure 4:**
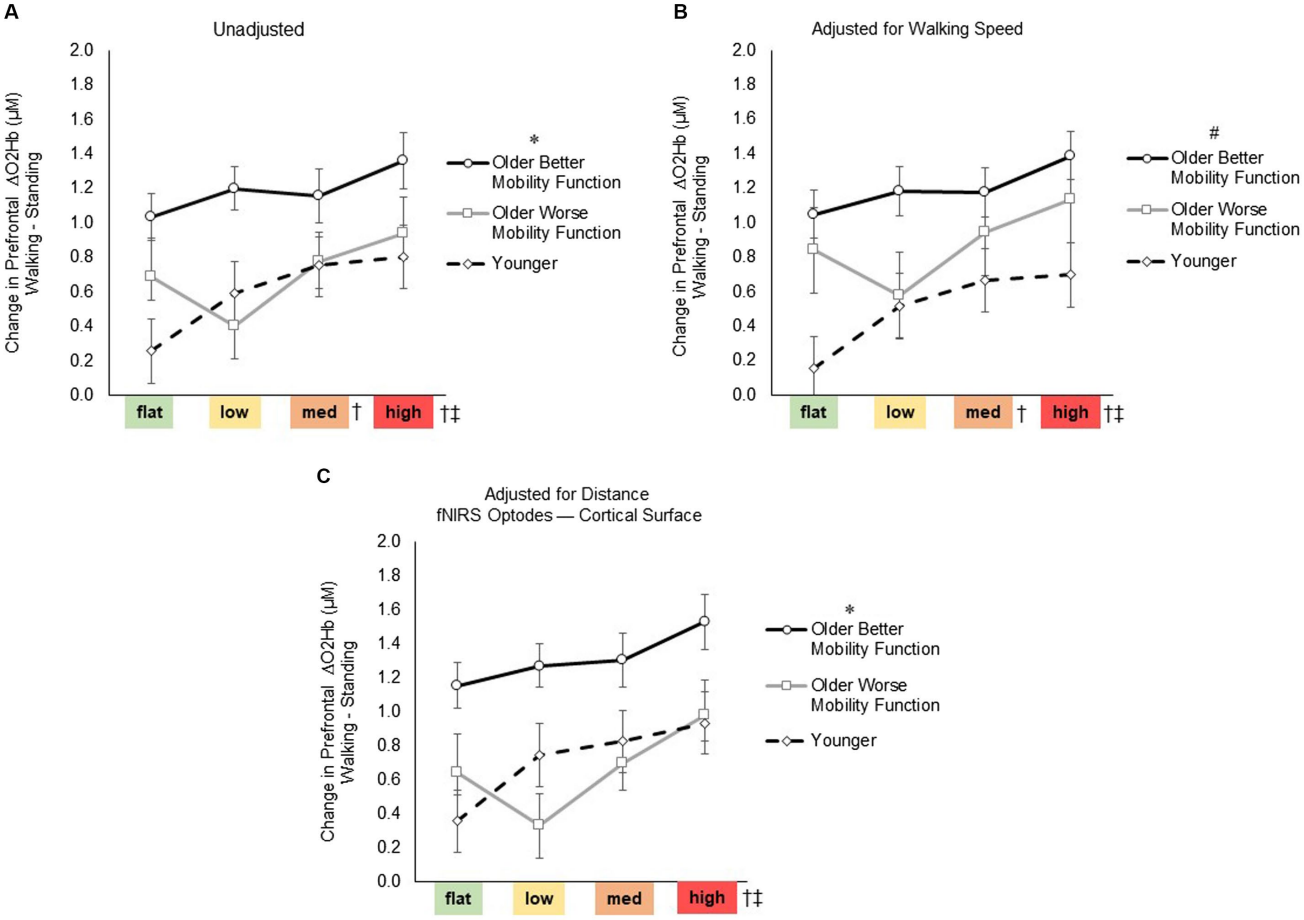
Task-related prefrontal cortical activation before **(A)** and after controlling for treadmill walking speed **(B)** and distance from the mean fNIRS F3 and F4 optodes to cortical surface **(C)**. Data represents the mean ± Standard error. * Represents significantly greater prefrontal cortical activation versus both younger adults and older adults with worse mobility function; #, significantly greater prefrontal cortical activation versus younger adults; †, significantly greater prefrontal cortical activation versus flat terrain; and ‡, significantly greater prefrontal cortical activation versus low terrain.

For prefrontal ΔO2Hb (Figure 4), the group by terrain interaction before (*F*_6, 247_ = 1.75, *p* = 0.116, η_p_^2^ = 0.05) or after adjusting for walking speed (*F*_6, 247_ = 1.72, *p* = 0.120, η_p_^2^ = 0.05) and distance from the mean fNIRS F3 and F4 optodes (*F*_6, 247_ = 1.34, *p* = 0.243, η_p_^2^ = 0.04) were not statistically significant, suggesting that the impact of task difficulty remained consistent across groups. However, a significant main effect of terrain was observed (*F*_3, 247_ = 8.04, *p* < 0.001, η_p_^2^ = 0.10; Figure 4A), indicating a significant difference among terrains in all pooled groups. Post-hoc tests revealed a significant increase in prefrontal ΔO2Hb for medium (*p* = 0.009) and high (*p* < 0.001) terrains compared with flat terrain, and for high terrain compared to low terrain (*p* = 0.002). Even after accounting for factors such as treadmill walking speed (*F*_3, 247_ = 7.98, *p* < 0.001, η_p_^2^ = 0.10; Figure 4B) and distance from the mean fNIRS F3 and F4 optodes to cortical surface (*F*_3, 247_ = 6.91, *p* < 0.001, η_p_^2^ = 0.11; Figure 4C), significant levels of unadjusted task-related ΔO2Hb persisted across terrains. Furthermore, a significant main effect of group was observed (*F*_2, 247_ = 5.84, *p* = 0.005, η_p_^2^ = 0.05; Figure 4A), indicating a notable difference among groups in all pooled terrains. *Post hoc* tests indicated overall increase in ΔO2Hb for older adults with better mobility function compared with both younger adults (*p* = 0.007) and older adults with worse mobility function (*p* = 0.034). However, there was no significant difference observed when comparing younger adults with older adults with worse mobility function (*p* = 0.696). Additionally, after accounting for treadmill walking speed (*F*_2, 247_ = 6.66, *p* = 0.003, η_p_^2^ = 0.06; Figure 4B), there was a general increase in the overall ΔO2Hb in older adults with worse mobility function, resulting in a lack of significant difference compared with older adults with better mobility function (*p* = 0.213). Furthermore, upon adjusting for distance from the mean fNIRS F3 and F4 optodes to the cortical surface (*F*_2, 247_ = 6.10, *p* = 0.044, η_p_^2^ = 0.07; Figure 4C), it appeared that the overall ΔO2Hb decreased in older adults with worse mobility function as indicated by a significant difference compared with older adults with better mobility function (*p* = 0.028). Additionally, after adjusting for the aforementioned variables, no significant differences were observed between older adults with worse mobility function and younger adults. Descriptive data is available in Supplementary Table S2. Full statistical results of prefrontal cortical activation adjusted for treadmill walking speed and distance from the mean fNIRS F3 and F4 optodes to cortical surface are provided in Supplementary material S3 Statistical Results.

#### 3.3.2 Prefrontal cortical activation by hemispheric sides

**Figure 5:**
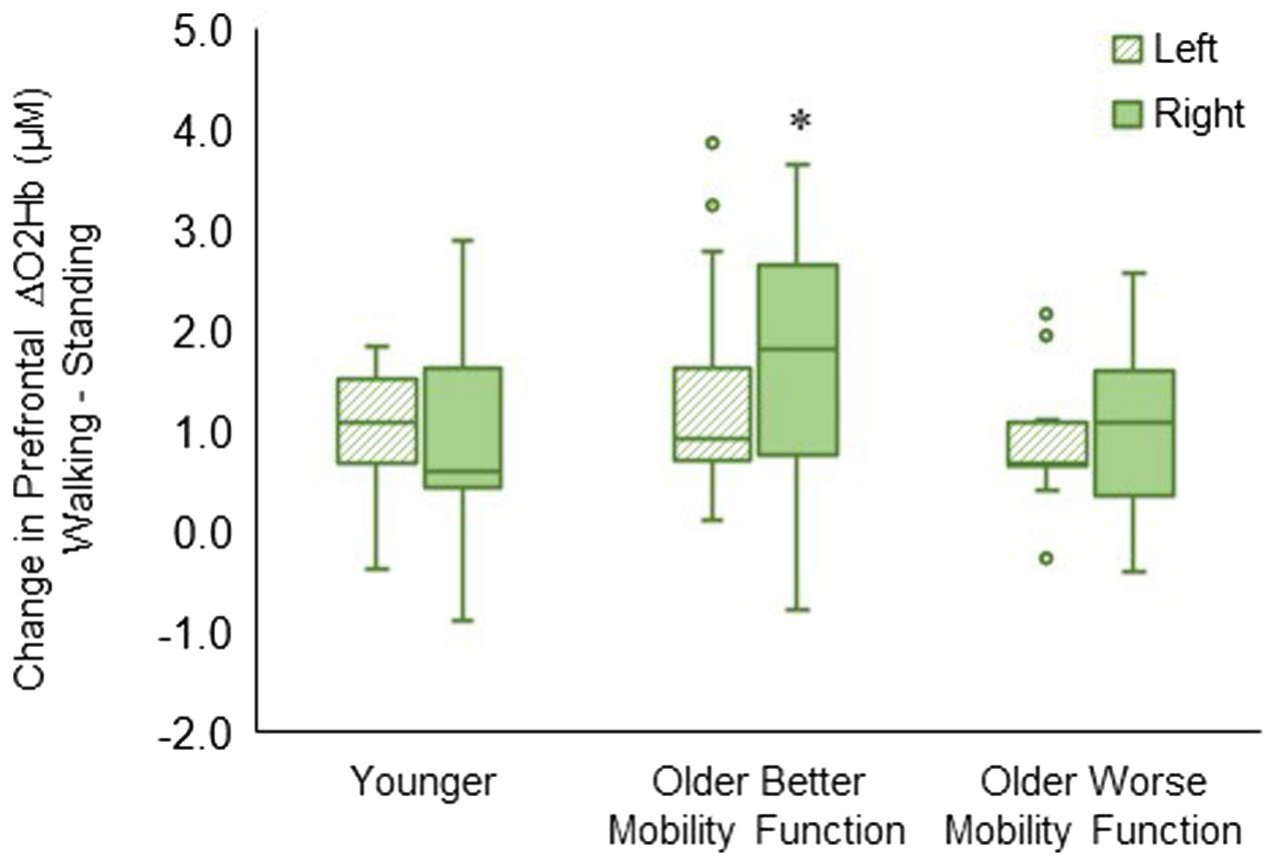
Box plots showing the distribution of hemisphere-related prefrontal cortex activation. Unfilled circles denote outliers. * Represents significantly greater for the right versus left hemisphere.

The group by hemisphere interaction was statistically significant (*F*_2, 494_ = 8.23, *p* < 0.001, η_p_^2^ = 0.04). *Post hoc* tests indicated that ΔO2Hb was greater in the right hemisphere than in the left hemisphere during walking (*p* < 0.001) in older adults with better mobility function. No statistically significant difference between left and right hemispheres was observed in older adults with worse mobility function (*p* = 0.096) and younger adults (*p* = 0.430) (Figure 5). The group by hemisphere by terrain interaction did not reach significance; therefore, no further analysis was pursued (*p* = 0.895). Descriptive data is available in Supplementary Table S4.

#### 3.3.3 Prefrontal cortical activation by recording site

**Figure 6:**
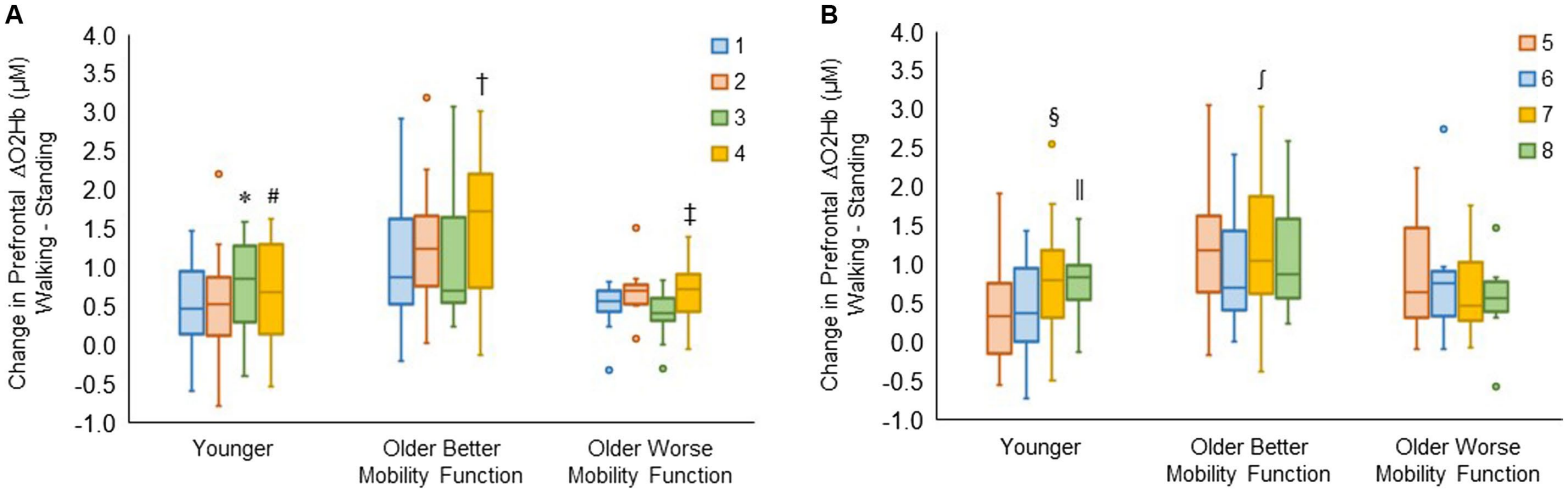
Box plots showing the distribution of channel-related prefrontal cortical activation in the right **(A)** and left **(B)** hemispheres. Unfilled circles denote outliers. * Represents significantly greater for channel 3 versus channels 1 and 2; #, significantly greater for channel 4 versus channel 2; †, significantly greater for channel 4 versus channel 1, 2, and 3; ‡, significantly greater for channel 4 versus channel 1 and 3; §, significantly greater for channel 7 versus channels 5 and 6; ‖, significantly greater for channel 8 versus channels 5 and 6; and ∫, significantly greater for channel 7 versus channel 6. The identical box plot colors for each channel in both **(A,B)** represent anatomically symmetrical positions between the right and left hemispheres.

The group by channel interaction for the right hemisphere reached statistical significance (*F*_6, 980_ = 3.90, *p* < 0.001, η_p_^2^ = 0.03). *Post hoc* tests for channels 1, 2, 3, and 4 revealed that ΔO2Hb was greater in channel 3 than in channel 1 (*p* = 0.009) and channel 2 (*p* = 0.006) and greater in channel 4 than in channel 2 (*p* = 0.039) in younger adults. In older adults with better mobility function, ΔO2Hb was greater in channel 4 than in channels 1 (*p* < 0.001), 2 (*p* = 0.006), and 3 (*p* < 0.001). Also, it was greater in channel 4 than in channels 1 (*p* = 0.029) and 3 (*p* = 0.008) in older adults with worse mobility function (Figure 6A).

The group by channel interaction for the left hemisphere was statistically significant (*F*_6, 983_ = 3.59, *p* = 0.002, η_p_^2^ = 0.02). *Post hoc* tests for channels 5, 6, 7, and 8 revealed that ΔO2Hb was higher in channel 7 compared with channels 5 (*p* = 0.026) and 6 (*p* = 0.008), and also higher in channel 8 compared with channels 5 (*p* = 0.020) and 6 (*p* = 0.006) in younger adults. It was observed that there was a higher ΔO2Hb in channel 7 compared with channel 6 (*p* = 0.004) in older adults with better mobility function. No significant differences were observed across channels in left hemispheres for older adults with worse mobility function (Figure 6B). The group by hemisphere by terrain interaction did not reach significance for either the right hemisphere (*p* = 0.996) or the left hemisphere (*p* = 0.989); therefore, no further analysis was undertaken. Descriptive data is available in Supplementary Table S5.

### 3.4 Task-related behavioral performance

#### 3.4.1 Walking cadence and its variability

**Figure 7:**
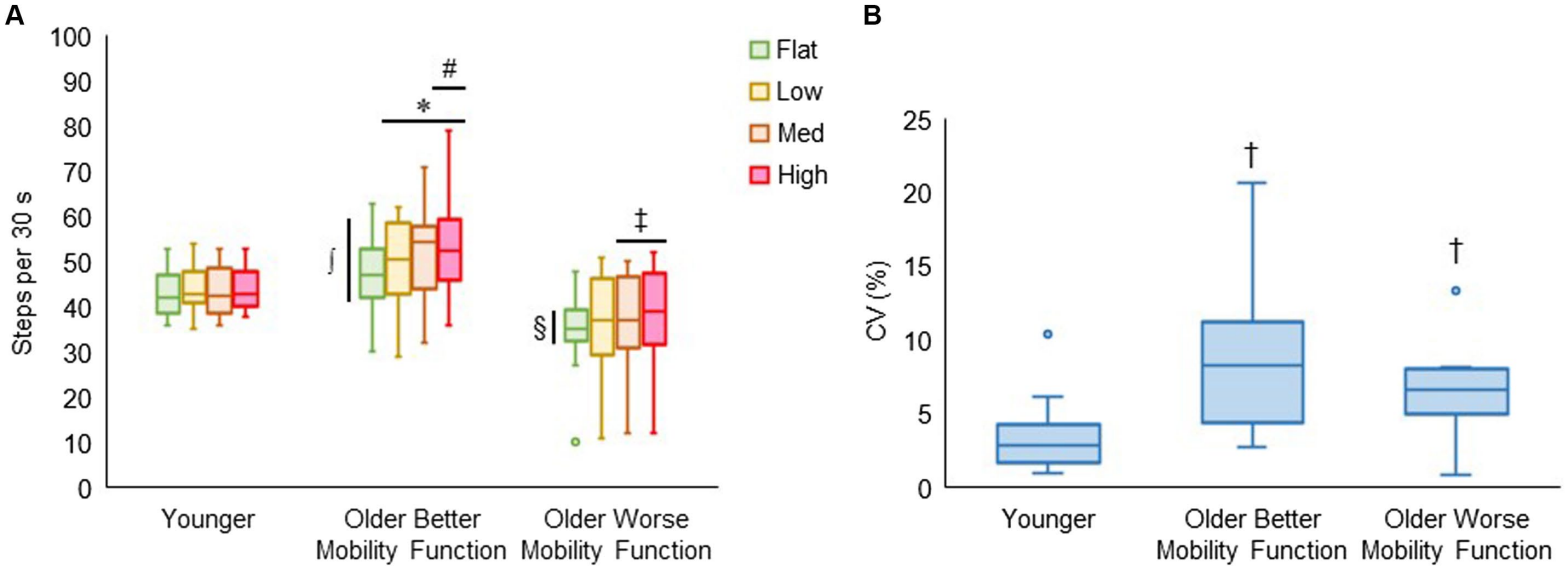
Box plots showing the distribution of walking cadence (steps per 30 s) during treadmill walking **(A)** and walking cadence variability (CV, coefficient of variation, %) across terrains **(B)**. Unfilled circles denote outliers. * Represents significantly greater number of steps for uneven terrains than flat terrain; #, significantly greater number of steps for high terrain than low terrain; ‡ significantly greater number of steps for medium and high terrains than flat terrain; ∫, significantly greater number of steps in older adults with better mobility function versus older adults with worse mobility function for all terrains and younger adults for medium and high terrains; and §, significantly fewer number of steps in older adults with worse mobility function versus the other two groups for flat terrain. † Represents significantly higher walking cadence variability in both groups of older adults versus younger adults.

Figure 7A displays walking cadence (steps per 30 s) during treadmill walking on each terrain by group. The group by terrain interaction was found to be significant (*F*_6, 176_ = 2.81, *p* < 0.014, η_p_^2^ = 0.12), indicating that the effect of task difficulty differed among groups, with a more pronounced differential effect observed in both groups of older adults compared with younger adults. *Post hoc* tests revealed that walking cadence was higher for low (*p* = 0.003), medium (*p* < 0.001), and high (*p* < 0.001) terrains compared with flat terrain, and significantly higher for high terrain (*p* = 0.025) compared with low terrain in older adults with better mobility function. Additionally, in older adults with worse mobility function, walking cadence was higher for medium (*p* = 0.027) and high terrain (*p* = 0.005) compared with flat terrain, while it remained consistent across all terrains in younger adults. Furthermore, older adults with better mobility function took more steps each 30 s of terrain walking for flat (*p* = 0.004), low (*p* < 0.001), medium (*p* < 0.001), and high (*p* < 0.001) terrains than older adults with worse mobility function. Also, older adults with better mobility function took more steps for medium (*p* = 0.013) and high (*p* = 0.009) terrains compared with younger adults. Older adults with worse mobility function compared with their peers (*p* = 0.004) and their younger counterparts (*p* = 0.049) took fewer steps only for flat terrain. Figure 7B shows that the coefficients of variation as a measure of walking cadence variability significantly differed between groups (*F*_2, 43_ = 7.30, *p* = 0.002), indicating that walking cadence variability was higher for older adults with better mobility function (8.35 ± 4.51%; *p* < 0.001) and older adults with worse mobility function (6.95 ± 3.61%; *p* = 0.015) compared with younger adults (3.43 ± 2.48%). Descriptive data are available in Supplementary Table S6.

#### 3.4.2 Participants’ perceived stability rating

**Figure 8:**
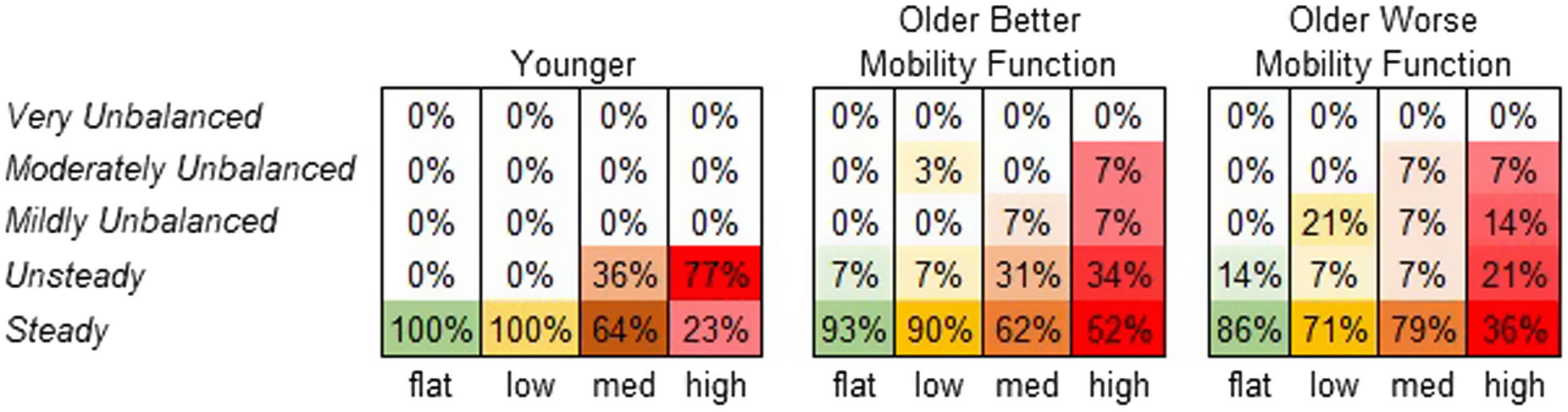
Perceived stability rating frequency by group. Darker shades of the specified color per condition indicate higher perceived stability rating frequency (%).

Figure 8 displays the frequency at which each terrain condition received a specific stability score from each participant group. A significant main effect of terrain on perceived stability rating was observed (χ^2^ = 65.7, *p* < 0.001). Compared with flat terrain, medium and high terrains were significantly more likely to be perceived as less stable (*p* < 0.001), and the odds of a less stable rating increased with the height of terrain unevenness (odds ratios: low = 2.2, medium = 10.0, and high = 31.1). However, a main effect of group was not significant, suggesting that perceived stability ratings did not significantly differ between groups (χ^2^ = 0.26, *p* = 0.876). The group by terrain interaction was not included in this model due to convergence issues. For further descriptive information, odds ratios were calculated for each group across terrains. Both older adults with better mobility function (odds ratios: low = 1.6, medium = 8.1, and high = 13.1) and older adults with worse mobility function (odds ratios: low = 3.8, medium = 2.3, and high = 21.2) were more likely to report feeling much less stable across terrains. Odds ratios for younger adults could not be obtained due to similar error variances across terrains (Vatcheva et al., 2016).

## 4 Discussion

Four key findings emerged from our analysis of age and mobility function effects on prefrontal cortical activation and task performance. First, walking on the terrain treadmill resulted in a significant increase in prefrontal cortical activation with escalating terrain unevenness, particularly on the highest terrain in all pooled groups. Second, older adults with better mobility function, compared with their younger counterparts and their peers, exhibited an overall greater level of prefrontal cortical activation. Third, hemispheric and regional differences in prefrontal regions were particularly evident in older adults with better mobility function. Fourth, the increasing difficulty of the terrain walking task was evident through an increase in walking cadence variability and a decrease in perceived stability, with both groups of older adults experiencing these effects more prominently than their younger counterparts. The results partially agreed with expectations based on the CRUNCH model, including higher prefrontal cortical activation for the older group with better mobility function. This implies compensation, but we did not see the expected plateau in brain activation as terrain unevenness increased. Furthermore, the older group with worse mobility function did not demonstrate the expected heightened prefrontal cortical activation compared with the other groups. Both physiological and methodological factors may have contributed to the unexpected findings.

### 4.1 Prefrontal cortical activation as a function of terrain unevenness during walking

The fNIRS results revealed heightened prefrontal cortical activation with increasing terrain unevenness in all groups. Notably, statistical significance was achieved in the medium and high uneven terrains when compared with the flat terrain, and the high uneven terrain when compared with the low uneven terrain. The findings of this study suggest that prefrontal cortical activation gradually increased with the escalating terrain unevenness similarly in all groups, as indicated by the absence of a significant interaction term for terrain by group. Our results are consistent with previous studies that explored various experimental contexts of walking with terrain modifications, such as navigating obstacles (Clark et al., 2014; Chen et al., 2017; Mirelman et al., 2017; Hawkins et al., 2018), traversing uneven terrain surfaces (Schack et al., 2022), and mental imagery of walking over obstacles and uneven surfaces (Wai et al., 2012; Allali et al., 2014). Those studies consistently reported heightened prefrontal cortical activation during complex walking tasks, indicating increased involvement of executive networks. Moreover, older adults often perform within a constrained functional range of brain activity (Clark, 2015). This constraint may stem from their tendency to allocate more cognitive resources during tasks with lower cognitive demands, eventually reaching a resource ceiling as task difficulty escalates (Reuter-Lorenz and Cappell, 2008). This functional range may be further constrained in older adults with cognitive and mobility impairments during the performance of a complex walking task (Hawkins et al., 2018; Chatterjee et al., 2020). However, in this study, while the constrained range was observed primarily in older groups, all groups exhibited a consistent increase in prefrontal cortical activation, indicated by a similar upward trend towards higher uneven terrains. Therefore, walking on the uneven terrain significantly contributed to the increase in prefrontal cortical activation.

Walking on the uneven terrain treadmill requires significant gait adjustments, potentially increasing the demand for executive control resources, especially in older participants (Fettrow et al., 2021b). For example, depending on the characteristics of the uneven surface, walking on an uneven treadmill surface can lead to a reduction in step time and length, resulting in a more cautious gait pattern with shorter steps (Voloshina and Ferris, 2015). This walking strategy may act as a way to maximize periods of double limb support to maintain stability. In this study, as terrain unevenness increased, older adults increased their walking cadence, particularly when compared with walking on flat terrain. In contrast, young adults maintained a consistent cadence across the four terrains. The difference between age groups implies a more adept walking ability for younger adults in diverse environments, including treadmill walking (Fettrow et al., 2021a). This is consistent with the finding of greater self-reported balance confidence in younger adults.

A recent study from our team (part of the larger Mind in Motion project) demonstrated that walking on an uneven terrain treadmill results in alterations in gait kinematic variability, especially in step duration and sacral excursion (Downey et al., 2022). The heightened variability was more notable in older adults with lower mobility function compared with those with higher mobility function, followed by younger adults. Our present finding reinforces this pattern, indicating that walking cadence variability increased with escalating terrain unevenness in both older groups, as opposed to the younger group. Thus, the altered terrain could heighten the difficulty of walking, potentially requiring greater cognitive demand to negotiate the irregular surfaces involving greater prefrontal cortical activation compared with walking on a flat surface. This aligns with previous studies that reported heightened prefrontal cortical activation during obstacle negotiation walking, coupled with increased gait variability in older adults (Mirelman et al., 2017; Nobrega-Sousa et al., 2020).

### 4.2 Age-related prefrontal cortical activation during terrain walking

Theoretical perspectives suggest that aging leads to a shift from a more automated control strategy (e.g., involving spinal cord networks) to a compensatory strategy of controlled processing via cortical regions involved in executive control (Clark et al., 2014; Clark, 2015). As expected, we observed heightened prefrontal cortical activation in older adults with better mobility function compared with younger adults in all pooled terrains. This observation aligns with numerous studies that consistently demonstrated greater prefrontal cortical activation during complex walking tasks in older adults, as opposed to younger adults (Holtzer et al., 2011; Fraser et al., 2016; Mirelman et al., 2017; Hawkins et al., 2018; Nobrega-Sousa et al., 2020). The heightened prefrontal cortical activation during walking likely reflects compensation (Cabeza et al., 2002; Reuter-Lorenz and Cappell, 2008). In the cognitive literature, heightened brain activation can be interpreted as neural compensation if it is associated with better performance or as neural dysfunction if it is linked with poorer performance (Cabeza et al., 2002; Fettrow et al., 2021b). Older adults with better mobility function may exhibit neural compensation to perform relatively well, displaying similar walking cadence variability even with their faster walking speed compared to their older mobility counterparts. Therefore, the increased prefrontal cortical activation observed in older adults with better mobility function supports this performance advantage.

We initially hypothesized that prefrontal cortical activation during walking on the terrain would be more pronounced for older adults with worse mobility function than their younger and better mobility counterparts. Contrary to our hypothesis, changes in prefrontal cortical activation did not significantly increase and differ across terrains when compared with the other experimental groups. It is possible that relatively less prefrontal cortical activation in older adults with worse mobility function may not be accompanied by preserved performance, suggesting neural dysfunction (Fettrow et al., 2021b). Nevertheless, this finding deviates from previous studies that reported heightened prefrontal cortical activation during tasks such as obstacle negotiation and dual-tasking walking in older adults with poorer mobility (i.e., slower walkers) (Chen et al., 2017), dual-task walking in older adults with neurological gait abnormalities (Holtzer et al., 2016), uneven terrain walking in older adults with lower limb amputation (Schack et al., 2022), and typical walking and obstacle negotiation in participants after stroke with lower motor impairment (Hawkins et al., 2018), all in comparison with controls.

The inconsistency in findings may be attributed to methodological differences in the task paradigm. In contrast to other studies that did not control walking speed across different conditions, our study employed a treadmill, maintaining a consistent speed across all terrains. All participants carried out the same terrain tasks on the treadmill at their individually determined walking speeds. However, treadmill walking speed was slower in older adults with worse mobility function than in their peers and younger counterparts in the current study, perhaps contributing to a lower absolute level of task complexity in older adults with worse mobility function. Indeed, after adjusting for treadmill walking speed, the magnitude of prefrontal cortical activation increased in older adults with worse mobility function (although statistically the activation level remained similar to that of young adults). Furthermore, we considered the impact of individuals’ cognitive capacity on task-related brain activation (Chatterjee et al., 2020). However, our cognitive MoCA assessment indicated a relatively high level of cognitive function in both groups of older adults, with no significant differences observed between the two older groups nor when compared to the younger group.

It is also plausible that older adults with worse mobility function may be less able to engage in compensatory brain activation, potentially due to frontal atrophy (Rombouts et al., 2003; Fjell et al., 2009). In this study, it was observed that older adults with worse mobility function exhibited a notably greater scalp-to-cortex distance compared with the other groups, indicating a potential higher degree of frontal atrophy (Seidler et al., 2010). After accounting for the distance, the level of prefrontal cortical activation in older adults with worse mobility function appeared to considerably decrease but remained statistically comparable to the unadjusted task-related prefrontal cortical activation. However, the effect size of the main group effect in the adjusted model was 22% higher compared with that in the unadjusted model. This implies that the distance from fNIRS optodes to the cortical surface, at least in part, affected our results.

### 4.3 Interpretation of age-related compensatory brain activity

The CRUNCH model, delineating compensatory neural mechanisms in response to increased task demands and the impact of aging, serves as a predictive framework for age-related compensatory brain activity (Reuter-Lorenz and Cappell, 2008). According to the fNIRS results, older adults with better mobility function exhibited heightened prefrontal cortical activation compared with their younger counterparts during walking particularly notable at lower terrain unevenness levels, consistent with observations in our previous studies during unobstructed typical walking (Clark et al., 2014; Hawkins et al., 2018; Chatterjee et al., 2020). This finding is likely in alignment with the CRUNCH model. However, as task demands increased, both groups of older adults continued to demonstrate increased levels of prefrontal cortical activation, rather than reaching a plateau in activation. In contrast, the prefrontal cortical activation in the younger group appeared to plateau considerably, possibly indicating no additional demand for executive resources as terrain unevenness increased. This contradicts the CRUNCH model’s prediction that brain activation in older adults reaches its peak and diminishes with increasing task demands, leading to insufficient processing and age-related decrements in performance with increasing task complexity (Reuter-Lorenz and Cappell, 2008; Schneider-Garces et al., 2010).

One plausible explanation for our findings is that walking on the uneven terrain did not pose a sufficient challenge for older adults to max out compensatory recruitment; in other words, the highest difficulty level did not push them to reach the compensatory recruitment ceiling. Nevertheless, older adults frequently reported feeling very or moderately unbalanced on high terrain. Specifically, older adults with better mobility function had 13 times the odds of feeling unstable compared with being on the flat surface, while older adults with worse mobility function had 21 times the odds (although there was no statistically significant difference between groups). This result suggests that, despite the likelihood of older adults reaching their compensatory recruitment potential during cognitive tasks (Mattay et al., 2006; Cappell et al., 2010), achieving the ceiling of available executive resources in a locomotor task such as uneven terrain walking may be challenging. Likewise, the young adults may have found the task to be relatively easy at the highest terrain, leading to a plateau in prefrontal recruitment due the lack of perceived complexity of the task.

Prior research has explored the CRUNCH model in diverse walking conditions, including typical walking vs. dual-task walking or obstacle crossing walking (Hawkins et al., 2018; Chatterjee et al., 2020; Hoang et al., 2022). In contrast, this current study tested the CRUNCH model under the same experimental context involving parametric manipulations of task difficulty while walking. Our findings indicate that walking on the terrain had differential effects on prefrontal cortical activation levels between younger and older adults, aligning, at least in part, with the CRUNCH model. Moreover, the heightened activation observed across all terrains in older adults may be viewed as a beneficial compensatory response during walking, rather than an age-related anomaly, serving to maintain a certain level of task performance.

Regarding another compensatory activation pattern in older adults, neuroimaging studies on cognitive aging propose that older adults engage different brain regions compared with younger adults when performing the same task (Cabeza, 2002; Reuter-Lorenz, 2002). A related possibility is that additional sites may be recruited because older adults encounter more difficulty and exert more effort on tasks compared with younger adults (Reuter-Lorenz, 2002). Notably, walking on the terrain activated processes that were lateralized in the right prefrontal regions, especially in older adults with better mobility function. In contrast, older adults with worse mobility function and younger adults exhibited bilateral prefrontal cortical activation during walking. Our findings are in line with previous studies investigating changes in prefrontal cortical activation during walking in older adults. For instance, Hoang and colleagues (Hoang et al., 2022) demonstrated greater prefrontal cortical activation in the right hemisphere during typical walking in older adults and dual-task walking in middle-aged adults, with no significant differences between the left and right hemispheres in young adults. In terms of walking speed, Greenfield and colleagues (Greenfield et al., 2023) observed that faster older walkers showed greater right hemisphere prefrontal cortical activation, while slower older walkers had no significant differences between hemispheres. In the present study, older adults with better mobility function, when executing the task at a faster walking speed, displayed additional prefrontal resource recruitment in the right hemisphere, indicating an increased need for executive resources as task demands heightened. This suggests that the right hemisphere in the prefrontal cortex may play a relatively more important role in cognitive aspects of terrain walking in older adults with worse mobility function.

A secondary analysis for this study aimed to investigate the regional variations in prefrontal cortical activation across fNIRS recording sites (channels) for each hemisphere. Younger adults exhibited larger increases in prefrontal cortical activation for channels 3 and 8, representing the dorsolateral prefrontal cortex in the right and left hemispheres, respectively. In contrast, older adults demonstrated larger increases in activation for channels 4 and 7, corresponding to the frontopolar cortex in the right and left hemispheres, respectively. The dorsolateral prefrontal cortex was notably activated during terrain walking for the younger group, while the frontopolar cortex areas showed notable activation in the older adult groups. This is supported by a recent study (Asahara et al., 2022) that investigated regional differences in prefrontal cortical activation during overground walking, in which both speed and cognitive demand were manipulated. In that study involving healthy young adults, the results indicate heightened activation of the dorsolateral prefrontal cortex when walking became more challenging (i.e., at a faster speed), and increased activation of the frontopolar cortex when the cognitive demand increased during walking (Asahara et al., 2022). Increased activation in the dorsolateral prefrontal cortex is commonly observed during typical walking in older adults (Hoang et al., 2022). This heightened activation is linked to an increase in voluntary executive command, attributed to a loss of automaticity in processes related to walking (Clark, 2015). Older adults may already exhibit a high level of dorsolateral prefrontal cortex activation during terrain walking. Consequently, in the current study, older adults with better mobility function may engage additional cognitive areas, such as the prefrontal cortex, while walking on the terrain. This engagement aids in mediating task-relevant cognitive operations and maintaining a certain level of task performance (Cabeza, 2002; Mansouri et al., 2017).

### 4.4 Limitations

Some limitations may challenge the interpretation of these data, prompting additional discussions. One limitation is that we measured only prefrontal cortex activation. Other brain regions, including (but not limited to) the parietal lobes, cerebellum, basal ganglia, and the brainstem, contribute to the neural control of human locomotion (Leisman et al., 2016). Our study identified lateralized activation and additional activation in the prefrontal cortex regions. Caution is advised when extending these findings to the broader functions of the prefrontal cortex regions, which entail substantial overlapping and interactive functions across these regions (Duncan, 2010). Currently, there is limited research employing fNIRS to validate the CRUNCH framework, primarily because CRUNCH has traditionally been assessed using fMRI. However, conducting fMRI during walking is impractical, leading us to employ fNIRS as an alternative method to assess cortical involvement during gait. Lastly, within the CRUNCH framework, our study design did not fully support differential load-dependent changes in prefrontal cortical activation in older adults compared with younger adults. However, as other fMRI studies explicitly acknowledged (Jamadar, 2020; Zapparoli et al., 2022; Van Ruitenbeek et al., 2023), tests of the CRUNCH model have not been consistently conducted under the same experimental context through parametric manipulations of task difficulty. Therefore, larger and better-controlled studies of the CRUNCH model predictions that span a wide range of task difficulty levels are important.

## 5 Conclusion

The present study showed that walking on terrain treadmill surfaces, with four parametric manipulations of task difficulty (flat, low, medium, and high), increased prefrontal cortical activation as terrain unevenness increased in younger and older adults. Compared with younger adults, older adults who had better mobility function demonstrated overall heightened prefrontal cortical activation across different terrain conditions. These findings indicate that hallmarks of the CRUNCH framework, which was developed for cognitive tasks, are also applicable to interpreting and understanding the implications of brain aging on the performance of complex walking tasks.

## Data availability statement

The raw data supporting the conclusions of this article will be made available by the authors, without undue reservation.

## Ethics statement

The studies involving humans were approved by the Institutional Review Board at the University of Florida. The studies were conducted in accordance with the local legislation and institutional requirements. The participants provided their written informed consent to participate in this study. Written informed consent was obtained from the individual(s) for the publication of any potentially identifiable images or data included in this article.

## Author contributions

JH: Data curation, Formal analysis, Investigation, Methodology, Software, Visualization, Writing – original draft. CL: Data curation, Formal analysis, Investigation, Methodology, Software, Writing – review & editing. SW: Data curation, Investigation, Methodology, Writing – review & editing. SC: Data curation, Investigation, Methodology, Software, Writing – review & editing, Formal analysis. AG: Data curation, Formal analysis, Software, Writing – review & editing, Methodology. CS: Methodology, Writing – review & editing. TM: Conceptualization, Funding acquisition, Investigation, Methodology, Project administration, Resources, Writing – review & editing. CH: Conceptualization, Funding acquisition, Investigation, Methodology, Project administration, Resources, Writing – review & editing. RS: Conceptualization, Funding acquisition, Investigation, Methodology, Project administration, Resources, Writing – review & editing. DF: Conceptualization, Funding acquisition, Investigation, Methodology, Project administration, Resources, Writing – review & editing. AR: Methodology, Validation, Writing – review & editing. DC: Conceptualization, Funding acquisition, Investigation, Methodology, Project administration, Resources, Supervision, Writing – review & editing.
