# Supplementary material for: Prefrontal cortical activity during uneven terrain walking in younger and older adults
Source: Front Aging Neurosci. 2024 May 3;16:1389488. doi: 10.3389/fnagi.2024.1389488 (PMC11099210; doi:10.3389/fnagi.2024.1389488)
Supplement: Supplementary file 1 [file Table_1.docx]

Supplemental Table S1. Descriptive data of overground walking speed

| Group\Dependent Variable | Condition | Mean | Std Dev |
| --- | --- | --- | --- |
| Younger adults | flat | 1.23 | 0.30 |
|  | low | 1.09 | 0.27 |
|  | med | 1.03 | 0.23 |
|  | high | 1.01 | 0.26 |
|  | treadmill | 0.73 | 0.17 |
| Older adults  better mobility function | flat | 1.05 | 0.28 |
|  | low | 0.93 | 0.28 |
|  | med | 0.84 | 0.25 |
|  | high | 0.78 | 0.28 |
|  | treadmill | 0.57 | 0.19 |
| Older adults  worse mobility function | flat | 0.72 | 0.17 |
|  | low | 0.56 | 0.22 |
|  | med | 0.50 | 0.23 |
|  | high | 0.44 | 0.19 |
|  | treadmill | 0.26 | 0.11 |

Supplemental Table S2. Descriptive data of unadjusted prefrontal cortical activation during performance of uneven terrain walking tasks

| Group\Dependent Variable | Condition | Mean | Std Dev |
| --- | --- | --- | --- |
| Younger adults | flat | 0.26 | 0.58 |
|  | low | 0.59 | 0.86 |
|  | med | 0.76 | 0.70 |
|  | high | 0.80 | 0.67 |
| Older adults  better mobility function | flat | 1.03 | 0.73 |
|  | low | 1.20 | 0.67 |
|  | med | 1.16 | 0.85 |
|  | high | 1.36 | 0.87 |
| Older adults  worse mobility function | flat | 0.69 | 0.82 |
|  | low | 0.40 | 0.67 |
|  | med | 0.78 | 0.51 |
|  | high | 0.94 | 0.75 |

Supplemental S3 Statistical Results

3.1. Task-related prefrontal cortical activation (ΔO_2_Hb), adjusted for treadmill walking speed.

The group by task interaction after controlling for walking speed was not statistically significant (F_6, 247_ = 1.72, p = 0.120, η_p_2 = 0.045), indicating that the effect of task difficulty did not differ between groups. After accounting for treadmill walking speed (**Figure 4B**), a significant main effect of terrain was observed (F_3, 247_ = 7.98, p < 0.001, η_p_2 = 0.104), signifying a significant difference in all pooled terrains. Post-hoc tests revealed a significant increase in ΔO2Hb for medium (p = 0.011) and high (p < 0.001) terrains compared to flat terrain, and for high terrain compared to low terrain (p = 0.001) in all pooled groups. Also, a significant main effect of group was observed (F_2, 247_ = 6.66, p = 0.003, η_p_2 = 0.058), indicating a notable overall increase in ΔO_2_Hb for older adults with better mobility function compared with younger adults (p = 0.001) in all pooled terrains. However, there was no significant difference observed when comparing older adults with worse mobility function to older adults with better mobility function (p = 0.256) and younger adults (p = 0.256).

3.2. Task-related prefrontal cortical activation (ΔO_2_Hb), adjusted for distance from the mean fNIRS F3 and F4 optodes to cortical surface.

The group by task interaction after controlling for walking speed was not statistically significant (F_6, 247_ = 1.34, p = 0.243, η_p_2 = 0.042), indicating that the effect of task difficulty did not differ between groups. After accounting for distance from the mean fNIRS F3 and F4 electrodes (**Figure 4C**), a significant main effect of terrain was observed (F_3, 247_ = 6.91, p < 0.001, η_p_2 = 0.109), signifying a significant difference in all pooled terrains. Post-hoc tests revealed a significant increase in ΔO2Hb for high terrain (p < 0.001) compared to flat terrain, and for high terrain compared to low terrain (p = 0.002). Also, a significant main effect of group was observed (F_2, 247_ = 6.10, p = 0.044, η_p_2 = 0.065), indicating a notable overall increase in ΔO_2_Hb for older adults with better mobility function compared with both younger adults (p = 0.012) and older adults with worse mobility function (p = 0.028) in all pooled terrains. However, there was no significant difference observed when comparing older adults with worse mobility function to younger adults (p = 0.854).

Supplemental Table S4. Descriptive data of prefrontal cortical activation by hemispheric side

| Group\Dependent Variable |  | Mean | Std Dev |
| --- | --- | --- | --- |
| Younger adults | left | 0.78 | 0.72 |
|  | right | 0.69 | 0.87 |
| Older adults  better mobility function | left | 1.12 | 0.79 |
|  | right | 1.50 | 1.03 |
| Older adults  worse mobility function | left | 0.62 | 0.75 |
|  | right | 0.84 | 0.81 |

Supplemental Table S5. Descriptive data of prefrontal cortical activation by channels

| Group\Dependent Variable | Right Side Channels | Mean | Std Dev | Left Side Channels | Mean | Std Dev |
| --- | --- | --- | --- | --- | --- | --- |
| Younger adults | *1* | 0.48 | 0.54 | *5* | 0.45 | 0.74 |
|  | *2* | 0.48 | 0.71 | *6* | 0.43 | 0.66 |
|  | *3* | 0.76 | 0.64 | *7* | 0.77 | 0.77 |
|  | *4* | 0.69 | 0.65 | *8* | 0.78 | 0.47 |
| Older adults  better mobility function | *1* | 1.10 | 0.81 | *5* | 1.23 | 0.88 |
|  | *2* | 1.25 | 0.75 | *6* | 0.93 | 0.66 |
|  | *3* | 1.11 | 0.81 | *7* | 1.25 | 0.89 |
|  | *4* | 1.50 | 0.90 | *8* | 1.11 | 0.69 |
| Older adults  worse mobility function | *1* | 0.54 | 0.66 | *5* | 0.88 | 0.78 |
|  | *2* | 0.73 | 0.56 | *6* | 0.76 | 0.72 |
|  | *3* | 0.47 | 0.64 | *7* | 0.74 | 0.77 |
|  | *4* | 0.84 | 0.71 | *8* | 0.62 | 0.67 |

Supplemental Table S6. Descriptive data for walking cadence (steps during a 30-s walking) by condition

| Group\Dependent Variable | Condition | Mean | Std Dev |
| --- | --- | --- | --- |
| Younger adults | flat | 42.44 | 5.32 |
|  | low | 43.22 | 5.40 |
|  | med | 42.98 | 5.33 |
|  | high | 43.38 | 4.28 |
| Older adults  better mobility function | flat | 46.31 | 8.50 |
|  | low | 50.19 | 9.32 |
|  | med | 52.25 | 10.78 |
|  | high | 53.10 | 10.84 |
| Older adults  worse mobility function | flat | 34.90 | 9.56 |
|  | low | 37.00 | 10.51 |
|  | med | 38.08 | 10.34 |
|  | high | 38.92 | 10.40 |
